# Non-communicable diseases in the Western Area District, Sierra Leone, following the Ebola outbreak

**DOI:** 10.12688/f1000research.18563.2

**Published:** 2019-11-19

**Authors:** Ibrahim Baimba Koroma, Dena Javadi, Katrina Hann, Anthony D Harries, Francis Smart, Thomas Samba

**Affiliations:** 1 Directorate of Policy, Planning and Information (DPPI), Ministry of Health and Sanitation (MoHS), Freetown, Sierra Leone; 2 Institute of Tropical Medicine, Antwerp, Belgium; 3 Sustainable Health Systems, Freetown, Sierra Leone; 4 International Union Against Tuberculosis and Lung Disease, Paris, France; 5 London School of Hygiene and Tropical Medicine, London, UK; 6 National Public Health Agency (NPHA), Ministry of Health and Sanitation (MoHS), Freetown, Sierra Leone

**Keywords:** SORT IT, operational research, Sustainable Development Goals, Universal Health Coverage, health systems

## Abstract

**Background:** Non-communicable diseases (NCDs) are the leading causes of morbidity and mortality in the world. During infectious disease outbreaks, such as the Ebola virus disease outbreak in West Africa from 2014-2015, the health system is often strained, and diagnosis, management and care of NCDs may be compromised. This study assessed numbers and distribution of NCDs in all health facilities in the Western-Area District, Sierra Leone, in the post-Ebola period (June–December 2015) comparing findings with the pre-Ebola (June–December 2013) and Ebola outbreak (June–December 2014) periods.

**Methods:** This was a cross-sectional study using secondary data from routine records of aggregate monthly NCD reports. Data were analysed using Open EPI and comparisons were made between the post-Ebola and pre-Ebola/Ebola periods using the chi-square test.

**Results:** There were 10,011 people reported with NCDs during the three six-month periods, with 6194 (62%) presenting at peripheral health units (PHU). Reported NCDs decreased during Ebola and increased post-Ebola, but did not recover to pre-Ebola levels. Hypertension cases remained fairly constant throughout being mainly managed at PHU. Numbers with diabetes mellitus generally stayed the same except for a significant post-Ebola increase in tertiary hospitals. Small numbers were reported with mental health disorders across all facilities in all time periods.

**Conclusion:** NCD reporting is recovering in the immediate post-Ebola period. Decentralization of NCD care is welcome and is an effective strategy for management as evidenced by hypertension. To be successful, this must be supported by strengthening other elements of the health system such as training of health workers, robust information and referral systems and reliable medicine supply chains.

## Introduction

Non-communicable diseases (NCDs) are the leading causes of morbidity and account for 70% of worldwide deaths, with the major burden felt in low- and middle-income countries (LMICs) ^1^. The problem is highlighted in the UN Sustainable Development Goals (SDGs), with SDG target 3.4 aiming to reduce premature mortality from NCDs by one-third between 2015 and 2030 ^2^. Progress is slow with poor health system responsiveness and weak monitoring capacity being important obstacles ^3^.

 In 2014–2015, Sierra Leone was hit by a devastating Ebola virus disease outbreak that highlighted the need for resilient health systems ^4, 5^. At that time, diagnosis, care and management of NCDs were in the process of decentralization from hospital to primary healthcare settings. In 2017, we reported on the burden and distributions of selected NCDs in a sample of health facilities in the Western-Area District, Sierra Leone, before and during the Ebola outbreak ^6^. There was a marked decline in reported NCD numbers in the Ebola compared with the pre-Ebola periods, and this was especially observed for hypertension (HTN) and diabetes mellitus (DM) and in secondary and tertiary hospitals compared with peripheral health units (PHUs). Several recommendations were made that included strengthening the diagnosis, management and monitoring of NCDs in all district health facilities.

We now have comprehensive reports from all the district facilities for six-month periods before Ebola (June–December 2013), during Ebola (June–December 2014) and post-Ebola (June–December 2015) and have taken the opportunity to assess what has happened to NCDs during this time. The study objectives were to assess reported NCD numbers, the distribution of NCDs and their stratification by health facility level in the Western-Area District in the post-Ebola period and compare findings with Ebola and pre-Ebola periods outlined above.

## Methods

### Data source

This was a cross-sectional study using secondary data from routine records. The setting and district monitoring system have been previously described ^6^. In the current study, however, all public health facilities in the Western-Area District, Sierra Leone, were included: 110 PHUs, 9 secondary and 4 tertiary hospitals. The study population included all people reported with cardiovascular disease (CVD), HTN, DM, mental health disorder (MHD) and tumours/cancer in June–December 2013, June–December 2014 and June–December 2015. Data sources were the aggregate monthly NCD reports available at the Directorate of Policy, Planning and Information, Ministry of Health and Sanitation. The NCD reporting form captures the following morbidities: cardiovascular diseases, diabetes mellitus, hypertension, mental health disorder, and tumour/cancer. Epilepsy is reported through a different process, and is not included in the NCD reporting form.

### Data analysis

Data were analysed using Open EPI version 3.03 (updated 2014/09/22), with comparisons made between the post-Ebola and pre-Ebola/Ebola periods using the chi square test. *P*<0.05 was considered to indicate a significant difference.

### Ethical approval

Ethics approval was obtained from the Sierra Leone Ethics and Scientific Committee (dated 14 December 2018) and the Ethics Advisory Group, International Union Against Tuberculosis and Lung Disease, Paris, France (EAG number 63/18). As aggregate data were used with no identifiers, the need for informed consent was waived by the ethics committees.

## Results

Altogether there were 10,011 people reported with NCDs during the three six-month periods, of whom 6194 (62%) were at PHU, 720 (7%) at secondary and 3097 (31%) at tertiary hospitals. Numbers with the five selected NCDs in the three different time periods are shown in Table 1. Key findings were: i) a decrease followed by an increase in NCDs in Ebola and post-Ebola periods with numbers not reaching pre-Ebola levels and this distribution was mirrored for HTN; ii) a large increase in DM in the post-Ebola period; iii) a decrease in CVD and tumours/cancer in the Ebola outbreak which continued to decline or stay unchanged in the post-Ebola period; and iv) small numbers of MHD in all three periods. These changes between the different periods were similar for males and females. 

**Table 1.** Numbers of people with non-communicable diseases in the Pre-Ebola, Ebola and Post-Ebola periods in the Western Area District, Sierra Leone: 2013 – 2015. Pre-Ebola: June to December 2013; Ebola: June to December 2014; Post-Ebola: June to December 2015.

Non-communicable disease	Individuals with non-communicable disease		
	Pre-Ebola, n	Ebola, n	Post-Ebola, n
Cardiovascular Disease	355	300	196
Diabetes Mellitus	282	230	457
Hypertension	3716	1851	2463
Mental Health Disorder	18	7	9
Tumour/Cancer	53	37	37
Total	4424	2425	3162

NCD distributions in the three health facility levels are shown in Table 2. Key findings were: i) 72% of reported HTN and 94% of reported MHD cases were at PHU, while 83% of reported CVD, 64% of reported DM and 94% of reported tumour/cancer cases were at tertiary hospitals; ii) the proportion of reported HTN cases was maintained over the three six-month periods at PHU in contrast to the hospital settings; iii) numbers with DM generally stayed the same except for a significant post-Ebola increase in tertiary hospitals; iv) those with CVD decreased significantly in the post-Ebola period in PHU and tertiary hospitals.

**Table 2.** People with non-communicable diseases by level of health facility in the Pre-Ebola, Ebola, and Post-Ebola periods, Western Area District, Sierra Leone: 2013–2015. Pre-Ebola: June to December 2013; Ebola: June to December 2014; Post-Ebola: June to December 2015.

Non-communicable disease	Individuals with non-communicable disease			
	Pre-Ebola, n (%)	Ebola, n (%)	Post-Ebola, n (%)	Total, N
*Peripheral Health Units*				
**Total**	**2819**	**1561**	**1814**	**6194**
Cardiovascular disease	88 (3) **	21 (1)	21 (1)	130
Diabetes mellitus	85 (3)	87 (6) *	69 (4)	241
Hypertension	2626 (93)	1446 (93)	1714 (94)	5786
Mental health disorder	17 (1)	7 (<1)	8 (1)	32
Tumour/cancer	3 (<1)	0 (<1)	2 (<1)	5
*Secondary Hospitals*				
**Total**	**409**	**104**	**207**	**720**
Cardiovascular disease	4 (1) *	6 (6)	8 (4)	18
Diabetes mellitus	46 (11) *	20 (19)	41 (20)	107
Hypertension	358 (88) **	78 (75)	157 (76)	593
Mental health disorder	0 (0)	0 (0)	0 (0)	0
Tumour/cancer	1 (<1)	0 (0)	1 (<1)	2
*Tertiary Care Hospitals*				
**Total**	**1196**	**760**	**1141**	**3097**
Cardiovascular disease	263 (22) **	273 (36) **	167 (15)	703
Diabetes mellitus	151 (13) **	123 (16) **	347 (30)	621
Hypertension	732 (61) **	327 (43) **	592 (52)	1651
Mental health disorder	1 (<1)	0 (0)	1 (<1)	2
Tumour/cancer	49 (4)	37 (5) *	34 (3)	120

The chi-square test was used to compare results of categorical variables in the post-Ebola period with the pre-Ebola and Ebola periods: * *P*< 0.05; ** *P*<0.001

## Discussion

The three most common NCDs during the study period were HTN, DM and CVD, with DM assuming greater importance in the post-Ebola period. Reasons are unclear, although DM has been predicted to increase dramatically in the next 10–20 years throughout sub-Saharan Africa ^7^. In all three six-month periods it was encouraging to see that HTN was predominately managed at the PHU as opposed to hospitals, suggesting that the process of decentralisation in this area is well underway. The management of DM could benefit from the same approach. The small number of individuals reported with MHD is of concern, especially given the high prevalence of anxiety, depression and post-traumatic stress disorder following the Ebola outbreak ^8^. This finding may reflect underreporting and a persisting treatment gap for MHD ^9, 10^.

The strengths of the study are the inclusion of all public health facilities, making this study representative of the district. Limitations include use of routine aggregate data (the accuracy of which could not be verified), the lack of data from the private sector and missing hospital data for 2016 and 2017, which prevented further assessment of the post-Ebola recovery period.

There are important policy recommendations from this study. It is clear that the majority of NCDs in the district are managed at the PHU; this move for decentralisation is in line with Sierra Leone’s 2017–2021 National Health Sector Strategic Plan ^11^. To ensure quality of care, however, the availability of medicines, diagnostic tools, a trained workforce, use of standardised NCD protocols and robust information systems must also be decentralised ^12^. Health facility reporting forms and processes must also be reassessed to improve consistency of case definitions and ensure complete and accurate records, without which resource management to strengthen NCD care is not possible.

In conclusion, this study shows that NCD reporting is recovering in the immediate post-Ebola period; however, the impact of reporting on service delivery and management requires further research. Decentralisation could be an effective and resilient strategy for management as evidenced by HTN. This encouraging momentum must be accompanied by health system strengthening—particularly for information systems—which will help move the country to universal health coverage and achievement of SDG3.4. 

## Data availability

Open Science Framework: Kamara_IbrahimK_SORTIT2_NCD_data 2019. https://doi.org/10.17605/OSF.IO/5Q3AX ^13^.

This project contains the number of cases of each disease for each time period, alongside a data dictionary.

The Sierra Leone Health Management Information Systems, the District Health Information System 2 (DHIS2), is accessible with a Ministry of Health and Sanitation login through https://sl.dhis2.org/. The Directorate of Policy, Planning, and Information (DPPI) can be contacted through Dr. Francis Smart ( drfsmart@gmail.com), Director, DPPI, MOHS, with an information request detailing the specific data request and purpose of use. Applicants will be asked to provide details of the reason for the request and details pertaining data request (such as data points, disaggregation, time period). In this case, data access would be granted to persons who request data for research purposes if they can provide appropriate ethical approval documentation.
