# Supplementary material for: Non-communicable diseases in the Western Area District, Sierra Leone, following the Ebola outbreak
Source: F1000Res. 2019 Nov 19;8:795. Originally published 2019 Jun 6. [Version 2] doi: 10.12688/f1000research.18563.2 (PMC6652098; doi:10.12688/f1000research.18563.2)
Supplement: Peer review history

# Peer review: Reviewer response for version 2

**DOI:** 10.5256/f1000research.23285.r56767

**Published:** 2019-11-21

**Authors:** Jeffery Edwards

**Affiliations:** 1 Department of Global Health, School of Public Health, University of Washington, WA, USA

I approve of the revised version.

I confirm that I have read this submission and believe that I have an appropriate level of expertise to confirm that it is of an acceptable scientific standard.

# Peer review: Reviewer response for version 1

**DOI:** 10.5256/f1000research.20320.r49599

**Published:** 2019-07-22

**Authors:** Priyakanta Nayak

**Affiliations:** 1 Field Epidemiology Training Program (FETP), Centers for Disease Control and Prevention, Dhaka, Bangladesh

This is a good study, a kind of situational analysis of NCD in Sierra Leone during Ebola episodes. The methodology is good and the investigator has done a good job in evaluating the NCD reports across the PHUs.

 The only concerned thing here is the conclusion. This is very generalized and did not necessarily explain about the artifacts. Its obvious that it would be increased awareness and increased surveillance/screening activities as a result of the endemic but the same can not be generalized to the overall scenario. I recommend more clarity in the conclusion.

I confirm that I have read this submission and believe that I have an appropriate level of expertise to confirm that it is of an acceptable scientific standard.

# Response

**Published:** 2019-10-26

**Authors:** Katrina Hann

**Affiliations:** Sustainable Health Systems, Sierra Leone

Zena Nyakoojo

 Senior Managing Editor

 F1000 Research Ltd.

 Middlesex House

 34-42 Cleveland Street

 London W1T 4LB

 UK

 Dear Zena Nyakoojo,

RE: Non-communicable diseases in the Western Area District, Sierra Leone, following the Ebola outbreak  [version 1; peer review: 2 approved]

 We have carefully read through the peer review and have revised our manuscript accordingly. We see the manuscript as improved as a result of this peer review process, and thank you for your support in taking the process to this stage.

 Please find for your consideration the following:

1. A point-by-point response to the comments and suggestions of the reviewers
2. A new revised version of the manuscript, in tracked changes
3. A new, revised version of the manuscript, no tracked changes.

 

 We hope that these modifications meet with your favourable review. Please do not hesitate to request any further changes.

 Kind regards,

 Ibrahim Baimba Koroma

 Directorate of Policy, Planning and Information (DPPI)

 Ministry of Health and Sanitation (MoHS)

 Freetown

 Sierra Leone

**Point-by-point response to the reviewer suggestions**

**Author Response to Reviewer Report for Version 1 from Priyankanta Nayak 22 Jul 2019**

 Thank you for your thoughtful review of the manuscript and your suggestions. We have responded as below.

**1. Reviewer comment:** **This is a good study, a kind of situational analysis of NCD in Sierra Leone during Ebola episodes. The methodology is good and the investigator has done a good job in evaluating the NCD reports across the PHUs.**

**Response:**

 Thank you very much.

 2. **Reviewer comment:** **The only concerned thing here is the conclusion. This is very generalized and did not necessarily explain about the artifacts. Its obvious that it would be increased awareness and increased surveillance/screening activities as a result of the endemic but the same can not be generalized to the overall scenario. I recommend more clarity in the conclusion.**

**Response:**

 Thank you for this thoughtful reflection. The conclusion only gave a snap shot of the latest developments of NCD reporting during the post-Ebola period and suggested strategies for the health sector to strengthen NCD reporting.

# Peer review: Reviewer response for version 1

**DOI:** 10.5256/f1000research.20320.r49596

**Published:** 2019-07-19

**Authors:** Jeffery Edwards

**Affiliations:** 1 Department of Global Health, School of Public Health, University of Washington, WA, USA

I appreciate the opportunity to review this well written and timely manuscript submitted by Koroma et al. It is certainly telling of the impact of the Ebola outbreak and the effects on the health system within Sierra Leone. Although it only uses aggregate data, the analysis is straight forward and highlights the struggles of providing/reporting on healthcare within a developing country. 

 I believe this manuscript is very much worth of indexing and will add significantly to the growing knowledge base surrounding healthcare system management in developing countries. I would suggest one minor correction and an area which could be further discussed, where there is a gap in services being at least reported, if not provided.

 First, the last sentence in the 3rd paragraph is incomplete and needs to be corrected.

 Secondly, it is extremely interesting the lack of reporting of mental health visits - across all three levels of care. Additionally, I am curious, as with other African countries, do mental health visits include epilepsy as well? If so, this is an interesting perspective that should be mentioned. 

 The authors mention that "the small number of individuals reported with MHD is of concern...especially... post Ebola..." I think this could be another key finding, especially if it includes those with epilepsy. The prevalence of MHD and epilepsy in developing countries is unknown and regardless of the Ebola outbreak, the tremendous gap in reporting found in this study suggests that they are under-recognized and under-treated. The authors might want to point this out and provide some recommendations for Sierra Leone and other countries with similar contexts.

I confirm that I have read this submission and believe that I have an appropriate level of expertise to confirm that it is of an acceptable scientific standard.

# Response

**Published:** 2019-10-26

**Authors:** Katrina Hann

**Affiliations:** Sustainable Health Systems, Sierra Leone

Zena Nyakoojo

Senior Managing Editor

F1000 Research Ltd.

Middlesex House

34-42 Cleveland Street

London W1T 4LB

UK

Dear Zena Nyakoojo,

RE: Non-communicable diseases in the Western Area District, Sierra Leone, following the Ebola outbreak  [version 1; peer review: 2 approved]

We have carefully read through the peer review and have revised our manuscript accordingly. We see the manuscript as improved as a result of this peer review process, and thank you for your support in taking the process to this stage.

Please find for your consideration the following:

1. A point-by-point response to the comments and suggestions of the reviewers
2. A new revised version of the manuscript, in tracked changes
3. A new, revised version of the manuscript, no tracked changes.

 

We hope that these modifications meet with your favourable review. Please do not hesitate to request any further changes.

Kind regards,

Ibrahim Baimba Koroma

Directorate of Policy, Planning and Information (DPPI)

Ministry of Health and Sanitation (MoHS)

Freetown

Sierra Leone

**Author Response to Reviewer Report for Version 1 from Jeffery Edwards 19 Jul 2019**

Thank you for your thoughtful review of the manuscript and your insightful suggestions. We have responded as below.

**1. Reviewer comment:** **I appreciate the opportunity to review this well written and timely manuscript submitted by Koroma et al. It is certainly telling of the impact of the Ebola outbreak and the effects on the health system within Sierra Leone. Although it only uses aggregate data, the analysis is straight forward and highlights the struggles of providing/reporting on healthcare within a developing country.** **I believe this manuscript is very much worth of indexing and will add significantly to the growing knowledge base surrounding healthcare system management in developing countries.**

**Response:**

Thank you very much.

**2. Reviewer comment:** **I would suggest one minor correction and an area which could be further discussed, where there is a gap in services being at least reported, if not provided. First, the last sentence in the 3rd paragraph is incomplete and needs to be corrected.**

**Response:**

Thank you for this direction. We have amended the sentence based on this feedback.

**3. Reviewer comment:** **Secondly, it is extremely interesting the lack of reporting of mental health visits - across all three levels of care. Additionally, I am curious, as with other African countries, do mental health visits include epilepsy as well? If so, this is an interesting perspective that should be mentioned.**

**Response:**

In Sierra Leone, epilepsy is considered as a neurological condition, but it is documented using a separate data capture form and reported into a different programme. Therefore, our study does not capture epilepsy results, and thus, we are limited in our ability to make conclusions on epilepsy specifically.

We have made a clarification on this point in the description of the NCD reporting form in the methods section.

**4. Reviewer comment:** **The authors mention that "the small number of individuals reported with MHD is of concern...especially... post Ebola..." I think this could be another key finding, especially if it includes those with epilepsy. The prevalence of MHD and epilepsy in developing countries is unknown and regardless of the Ebola outbreak, the tremendous gap in reporting found in this study suggests that they are under-recognized and under-treated. The authors might want to point this out and provide some recommendations for Sierra Leone and other countries with similar contexts.**

**Response:**

Thank you for this insight. We have added an additional point on underreporting and the treatment gap in the discussion.
